# Ursolic acid enhances the effect of exercise training on vascular aging by reducing oxidative stress in aged type 2 diabetic rats

**DOI:** 10.1002/fsn3.3105

**Published:** 2022-10-17

**Authors:** Masume Kazemi Pordanjani, Ebrahim Banitalebi, Mehrdad Roghani, Roohullah Hemmati

**Affiliations:** ^1^ Department of Sport Sciences Shahrekord University Shahrekord Iran; ^2^ Department of Physiology, Neurophysiology Research Center Shahed University Tehran Iran; ^3^ Department of Biology Shahrekord University Shahrekord Iran

**Keywords:** cellular senescence, diabetic angiopathies, exercise training, Sirtuin 1, ursolic acid

## Abstract

Ursolic acid (UA) mediates the vasorelaxant activity via nitric oxide (NO) release, and upregulation of endothelial nitric oxide synthase (eNOS) in endothelial cells (ECs) in disease conditions with increased oxidative stress (OS). The present study aimed to reflect on the impact of 8 weeks of a combination of UA supplementation and resistance/endurance training in old male Wistar rats having a high‐fat diet and/or low‐dose streptozotocin‐induced type 2 diabetes (HFD/STZ‐induced T2D), with an emphasis on Sirtuin 1 (SIRT1)–endothelial nitric oxide synthase (eNOS) axis and OS indices in their aortic tissues. A total number of56 21‐month‐old male Wistar rats with HFD/STZ‐induced T2D were randomized into seven groups (n = eight animals per group): (1) sedentary old nondiabetic (Control [C]); (2) sedentary HFD/STZ‐induced T2D (Diabetic [D]); (3) sedentary HFD/STZ‐induced T2D plus UA (Diabetic + Ursolic Acid [DU]); (4) endurance‐trained HFD/STZ‐induced T2D (Diabetic + Endurance Training [DE]); (5) resistance‐trained HFD/STZ‐induced T2D (Diabetic + Resistance Training [DR]); (6) endurance‐trained HFD/STZ‐induced T2D plus UA (Diabetic + Endurance Training + Ursolic Acid [DEU]); and (7) resistance‐trained STZ–diabetic plus UA (Diabetic + Resistance Training + Ursolic Acid [DRU]) rats. The ladder‐based resistance training group performed the ladder resistance training at 60% of the maximum voluntary carrying capacity (MVCC), 14–20 climbs in each session, with a one‐min rest between each two trials, 5 days a week. The treadmill‐based endurance exercise training protocol consisted of repeated bouts of high‐ and low‐intensity training with 60–75% maximal running speed and 30%–40% maximal running speed in the course of 8 weeks, respectively. The animals in the supplement groups also took 500 mg of UA/kg of high‐fat diet/day, resulting in a daily UA intake of approximately 250 mg UA per kg of body weight rat/day. The resistance/endurance training plus the UA consumption could partially reverse the levels of malondialdehyde (MDA), nitric oxide (NO), as well as total antioxidant capacity (TAC). It was concluded that oral 0.5% UA supplementation can prevent vascular aging biomarkers in a HFD/STZ‐induced T2D model. Further studies are also required to clarify how chronic consumption of UA with/without training protocols reverses vascular aging process.

## Graphical abstract

Ursolic acid (UA) supplementation affects vascular aging biomarkers. UA supplementation plus resistance/endurance training did not affect vascular aging biomarkers.

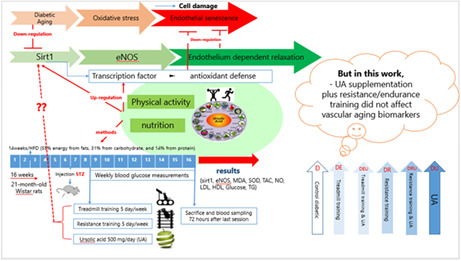

## 1 INTRODUCTION

Both aging and diabetes are two well‐known risk factors associated with vascular aging/senescence, so that diabetes increases the aging process by augmenting the severity of the loss of autonomic functions (Pararajasingam et al., 2021; Petrofsky & Lee, 2005). The majority of the mechanisms associated with vascular aging are thus correlated with advancing age, which overlap with the mechanisms present in diabetes (Assar et al., 2016).

Among diabetic vascular dysfunction biomarkers, one of the most robust and promising is SIRT 1 (a histone deacetylase belonging to the family of Sirtuins and a class of nicotinamide adenine dinucleotide [NAD^+^]‐dependent enzymes with multiple metabolic functions) (Kume et al., 2010; Meng et al., 2020; Ministrini et al., 2021; Orimo et al., 2009). Besides, SIRT1–endothelial nitric oxide synthase (eNOS) axis (Ota et al., 2010), oxidative stress (OS), malondialdehyde (MDA), lipid profile, as well as total antioxidant capacity (TAC) and superoxide dismutase (SOD) (Puca et al., 2013) are among the indices of endothelial–vascular function impairment due to aging and diabetes (Assar et al., 2016). However, the mechanisms underlying the changes in the SIRT1–eNOS axis mediated by aging and diabetes in endothelial cells (ECs) remain obscure (Zhang et al., 2017). It seems that the SIRT1–eNOS axis can account for the above‐mentioned mechanisms in case of aging and diabetes coexistence and influence of vascular functions (Ota et al., 2010; Zhang et al., 2017).

Modifiable lifestyle factors for healthy aging, such as increased physical activity and exercise training, have been advocated to improve diabetic vascular dysfunction (Lee et al., 2018; Qiu et al., 2018). Recently, evidence has illustrated the impact of resistance/endurance training on the SIRT1–eNOS axis and OS indices (Donniacuo et al., 2019; Radak et al., 2020). Ferrara et al. (2008) had reported that 6 weeks of an endurance‐type training had elevated SIRT1 activity in the heart of aged rat models (Ferrara et al., 2008).

Multimodal interventions (namely, training plus supplement intake) may boost the efficacy of training programs for less‐sensitive individuals such as aged cases with diabetes. So, Ursolic acid (UA, 3 *β*‐hydroxy‐urs‐12‐en‐28‐oic acid) is a major component of various traditional Chinese medicinal herbs, plants, fruits, and foods such as Fructus mume, Gardeniae fructus, Fructus ligustri lucidi, Hedyotis diffusa Willd and apple peel (Cargnin & Gnoatto, 2017; Sun et al., 2020), and is well known to have a wide range of biological functions, including antioxidant, neuroprotection, hepatoprotection, regulating blood glucose, and anti‐inflammatory activities (Agrawal et al., 2020; Liu, 1995; Sun et al., 2020). In this regard, it has been shown that UA mediates the vasorelaxant activity via nitric oxide (NO) release in the aorta tissue (Aguirre‐Crespo et al., 2006), and upregulation of eNOS and downregulation of Nox4 (nicotinamide adenine dinucleotide phosphate (NADPH) Oxidase 4) in human ECs in disease conditions with increased OS (Steinkamp‐Fenske et al., 2007). Schwaiger et al. (2011) illustrated the anti‐inflammatory effects of UA and supported the use of traditional herbal food which is rich in UA for the treatment of chronically inflammatory processes (Schwaiger et al., 2011). Recently, Bakhtiari et al. (2018) had revealed that UA had elevated SIRT1 activities in molecular docking and experimental studies (Bakhtiari et al., 2018).

Several lines of evidence have further referred to enhanced SIRT1 content following an increase in the rate of training (Civitarese et al., 2007; Corbi et al., 2012; Fulco et al., 2003; Lagouge et al., 2006; Suwa et al., 2008); nonetheless, various outputs have failed to demonstrate changes in the gene expression of SIRT1 based on training modality (Huffman et al., 2008). Moreover, variations have been observed in training intensity, volume, duration, SIRT1 content measurement, as well as respective tissues. In this line, Chan et al. (2018) had reflected on the expression of SIRT1 in aortic ECs after a 14‐week endurance training program. Chronic exercises had thus shown a descending trend in the protein expression level of SIRT1 in hyperhomocysteinemia‐induced aortic endothelial oxidative injury (Chan et al., 2018).

Based on the established and pivotal roles of SIRT1–eNOS axis in regulating endothelial function, early vascular aging (Man et al., 2019) and inhibition of OS (W. Zhang et al., 2017), and effects of natural bioactive compound, i.e. UA against endothelial dysfunction and increased OS (Li et al., 2016), it was first hypothesized that a combination of UA and resistance/endurance training, compared with no exercise training/placebo in the control group, would differently change the SIRT1–eNOS axis in high‐fat diet and/or low‐dose streptozotocin‐induced type 2 diabetes (HFD/STZ‐induced T2D) in old rats. Subsequently, it was assumed that OS‐induced vascular aging variables (OS, MDA, lipid profile, TAC, and SOD) might be directly sensitive to a combination of UA supplementation and resistance/endurance training HFD/STZ‐induced T2D in old rats. Therefore, to develop an optimal combination of pharmacological and physical activity treatments for the amelioration of diabetic vascular dysfunction, in this work we examined the minimal and optimal doses of two types of exercise training and UA consumption on vascular aging‐related variables in old male Wistar rats with high‐fat diet and or low‐dose streptozotocin‐induced type 2 diabetes (HFD/STZ‐induced T2D) with regard to the SIRT1–eNOS axis and OS biomarkers.

## 2 MATERIALS AND METHODS

### 2.1 Animal

The local Ethics Committee for Laboratory Animals of Shahrekord University (IR.SKU.REC.1399.001, ethics.research.ac.ir) approved the study. All methods were performed in accordance with the relevant guidelines and regulations. The experiment was conducted in accordance with the ARRIVE (Animal Research: Reporting of In Vivo Experiments) guidelines (Animal Research; Karp et al., 2015) for the care and use of research animals.

A total number of 56 21‐month‐old male Wistar rats, weighing 427 ± 44 g, were accordingly purchased from the Pasteur Institute, Tehran, Iran, and then maintained at a temperature‐controlled facility (20–22°C) with 40%–70% humidity, 12‐h light/dark cycle, as well as free access to a commercial standard pellet chow diet and water during the experiments. As the rats became familiarized with the laboratory conditions, they were randomized into seven groups (n = eight animals per group): (1) sedentary old nondiabetic (C); (2) sedentary HFD/STZ‐induced T2D (D); (3) sedentary HFD/STZ‐induced T2D plus UA (DU); (4) endurance‐trained HFD/STZ‐induced T2D (DE); (5) resistance‐trained HFD/STZ‐induced T2D (DR); (6) endurance‐trained HFD/STZ‐induced T2D plus UA (DEU); and (7) resistance‐trained STZ‐diabetic plus UA (DRU) rats.

### 2.2 HFD/STZ‐induced T2D

**FIGURE 1:**
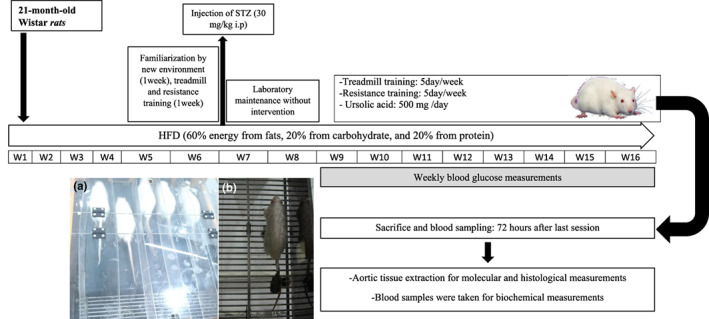
Study design, following 16‐weeks (a) treadmill and ladder climbing training (b).

As demonstrated in the protocol presented by Zhang et al. (2008) and Liu et al. (2013), HFD/STZ‐induced T2D was stimulated. Therefore, the animals in the control group were fed with a standard rodent‐chow diet. Whereas the animals in the HFD groups were placed on a high‐fat diet; that is, HF; energy from fats (60%), carbohydrates (20%), as well as protein (20%) = 5.21 kcal/g. Then, an 8‐week diet was considered for maintaining the groups. In the course of the fourth week, the HFD/STZ‐induced T2D group was treated with low‐dose STZ (Sigma‐Aldrich, CAS Number: 1888366‐4). In the next step, the low‐dose STZ, that is, 30 mg/kg dissolved in 0.1 M sodium‐citrate buffer at a pH of 4.4, was injected to the animals intraperitoneally. The level of blood glucose was also tested after the first week with a blood glucose meter (Roche). Accordingly, the animals with blood glucose levels <16.7 mmol/L were injected with STZ (30 mg/kg) for the second time. After that, the nondiabetic group was injected with sodium‐citrate buffer (as a vehicle) (0.25 ml/kg). It is notable that the mentioned diets were maintained at the postinjection stage. After 4 weeks, each rat with the blood glucose concentration >16.7 mmol/L was regarded to be diabetic and thus was chosen for additional investigations (de Bem et al., 2018; Qian et al., 2015).Subsequently, HFD/STZ‐induced T2D old male Wistar rats were fed with the high‐fat diet (55% fats, 31% carbohydrates, and 14% protein). The sedentary old nondiabetic animals (C group) also received a standard diet (10% fats, 75% carbohydrates, and 15% protein) throughout the study. The HFD plus UA (250 mg UA per kg of body weight rat/day) was further prepared by mixing 500 mg of UA per kg of HFD (0.5% UA plus HFD, Royan Company; Li et al., 2014; Figure 1). It should be noted that the high‐fat diet plus UA was prepared at the three‐day interval to avoid the oxidation of fat or other compounds (Knowledge‐Based Company, Healthy‐Aging Supplement 9870). The daily mean amount of the food intake was calculated as a difference between the amount of the remained food and the total one provided, divided by the number of days and rats in cages. Since the energetic values between the diets differed, the use of food in grams was converted into the caloric intake (Tófolo et al., 2019) and finally weekly body weight for each rat was measured during the investigation (Jayaprakasam et al., 2006).

### 2.3 The supplement intake

The animals in the supplement groups also took 500 mg of UA/kg of high‐fat diet/day, resulting in a daily UA intake of approximately 250 mg UA per kg of body weight rat/day.

### 2.4 Exercise testing and exercise training

To evaluate the maximal running speed, a ten three‐min phase running test on a rodent treadmill was used. According to Leandro et al. (2007) the initial running speed test was equal to 0.3 km/h and then speed elevated by 0.3 km.h^−1^ per 3 min (the slope was equal to 0%). Therefore, in case of the rats' inability to continue the running in all phases of the experiment, the speed at that phase was considered as the maximal running speed (Leandro et al., 2007).To evaluate the determination of maximum voluntary carrying capacity (MVCC), the animals were let to be familiarized with a vertical climbing model (110 cm, 2 cm grid, 85° incline) without overloading (Singulani et al., 2017). Following a 72‐h familiarization, all the animals were also tested to assess the respective MVCC. However, all of them carried a load of 75% of the body weight to the top of the ladder (namely, a house chamber) for the first climb. Then, the rats were allowed to rest for 120 s. After that, some weight increments of 30 g were added till the load did not let the rats climb the whole length of the ladder. It is noteworthy that the given process was iterated, so that the animals were not voluntarily capable of climbing the entire length of the ladder on three subsequent efforts. Finally, the MVCC was viewed as the successfully greatest load over the whole length of the ladder (Singulani et al., 2017).

In the resistance training protocol, the rats became familiarized with the apparatus by climbing it without the weights attached to their tails. Moreover, the weights were then fastened to the animals' tails. In crucial circumstances, a physical stimulus with finger pinching on the rats' tails was further employed as a stimulus for initiating the climbing movements. Upon arrival of the animals in a housing chamber, they were let to rest for 2 min. Then, the procedure was iterated till the animals could voluntarily climb for three consecutive attempts (Farsani et al., 2019). The resistance training group also performed the ladder resistance training at 60% of the MVCC, 14–20 climbs in each session, with a one‐min rest between each two trials, 5 days a week (Macedo et al., 2014). Finally, the group with diabetes was restricted to do any physical exercise training beyond the normal cage activities.

Within the 8‐week endurance training, the animals were exposed to endurance training programs (Nourshahi et al., 2012).

Then, the training intensity was calculated by maximal running speed. At the beginning of the endurance training, the animals were trained at 40–50% of maximal running speed for 5 min and 0% incline for warm‐up. The endurance training protocol consisted of repeated bouts of high‐ and low‐intensity training, 2 min of running with 60% maximal running speed in the course of the first week, 65% maximal running speed in the course of the second week, 70% maximal running speed in the course of the third week, and finally 75% maximal running speed in the course of the fourth week to the completion of the training time. Moreover, low‐intensity interval bouts involved two‐min running with 40% maximal running speed from the first week to the end of the third week and 30% maximal running speed from the onset of the fourth week to the completion of the eighth week. At the end, the number of the high‐intensity interval bouts increased from two to eight repeats from the first to the end of the eighth week **(**Figure 1 **).**

### 2.5 Blood sample and collection of aortic tissue

The rats were weighed and anesthetized through intraperitoneal administration of a mixture of 90 mg/kg ketamine and 10 mg/kg xylazine (Sigma Chemical Co). The animals were then sacrificed 48 h following the final resistance/endurance training session. The thoracic aortas were then gently dissected out and immediately immersed in liquid nitrogen.

### 2.6 Western blotting

According to the research design, Western blotting was run on the homogenates of the aortic tissues. Briefly, a pestle was employed to powder about 50 mg of the aortic tissue piece in liquid nitrogen and then lysed with a 1 ml of phosphate‐buffered saline (PBS). Moreover, this buffer was complemented with a protease inhibitor cocktail consisting of pepstatin, leupeptin, aprotinin, antipain, as well as chymostatin (5 μg/ml each) and rotated for 20 min at 4°C. In addition, they were centrifuged at 12,000 × *g* at a temperature of 4°C for 10 min. Hence, this supernatant was gathered and kept at −80°C. The total protein content of the tissue extract was correspondingly determined by the Bradford method (Bio‐Rad Laboratories) and spectrophotometric measurements (Jenway™ 6305 UV/Visible Spectrophotometer, Bibby Scientific Ltd.). The proteins were then isolated through the sodium dodecyl sulfate polyacrylamide gel electrophoresis (SDS‐PAGE) technique (10 μg protein loaded in the wells) and transported electrophoretically over the polyvinylidene fluoride or polyvinylidene difluoride (PVDF) membranes. It should be noted that the nonspecific binding was further obstructed by a two‐h incubation of the membrane in 5% (w/v) nonfat dry milk in the Tris‐buffered saline (TBS) at a power of hydrogen (pH) of 7.5. After that, the incubation of the blots was done for 2 h at the room temperature or overnight at a temperature of 4°C with the primary antibodies; that is, anti‐eNOS (Santa Cruz, USA, sc‐376751), anti‐SIRT1 (Santa Cruz, USA, sc‐74465), as well as anti‐*β*‐actin (Santa Cruz, USA, sc‐81178) 1:500, diluted in an antibody buffer consisting of 1% (w/v) nonfat dry milk in TBS and Polysorbate 20 (Tween 20) (0.05% [v/v] in the TBS). Afterwards, they were washed three times with Tris‐buffered saline with Tween 20 (TBS‐T) and consequently incubation was fulfilled for 1 h with a secondary antibody, that is, goat antirabbit (immunoglobulin G (IgG)) (Santa Cruz, USA, sc‐2004) 1:5000, in the antibody buffer. Next, the blots were developed to visualize by means of the enhanced chemiluminescence (ECL) detection kit (Pierce, Catalog Number: 32106). Finally, anti‐*β*‐actin was employed as one of the loading controls. The band intensity on immunoblots was further quantified via densitometry using the Image Studio Lite software. All other reagents and chemicals were obtained from Sigma‐Aldrich.

### 2.7 Plasma glucose, insulin, and lipid measurement

In this step, fasting blood glucose (FBG) in the tail vein blood sample was measured at the beginning of the experiment through a standard glucometer (Accu‐Chek Active) to ensure that the animals had euglycemia. Following the HFD/STZ‐induced T2D, blood glucose level was measured to assess the initiation of hyperglycemia (FBG 200 mg/dl). Moreover, the enzyme‐linked immunosorbent assay (ELISA; Cat No: 10‐1250‐01, Mercodia) was utilized to measure the plasma insulin levels in the rats that had been fasted for 4 h. In addition, total concentrations of serum cholesterol, high‐density lipoprotein cholesterol (HDL‐C), and triglycerides (TG) were assessed by enzymatic methods using commercially available diagnostic kits (CH201, CH203, and TR210, respectively), based on the company's directions (Randox Laboratories Ltd., Crumlin, Co.).

### 2.8 Antioxidant activity

Malondialdehyde (MDA), SOD, and NO were measured with the use of ZellBio assay kit (ZellBio GmbH), based on the company's directions, that take a colorimetric method which is a simplified process by the lab techniques. So for TAC, a fluorimetry method is taken with the use of Randox assay kit.

### 2.9 Data analysis and statistics

In this study, only data from diabetic groups were included in statistical analysis, and data from healthy control group were not included in the comparison between groups. In the graphs drawn, the data of the healthy control group were placed next to the data of the diabetic groups only to show, without involving them in the statistical comparison. Data were expressed as mean ± standard error of the mean. Repeated‐measures analyses of variance (ANOVAs) were used to investigate changes in the three levels for maximal running speed and MVCC (i.e., weeks one, four, and eight), five levels for blood glucose (namely, weeks one, two, four, six, and eight), and eight levels for body weight (namely, weeks one, two, three, four, five, six, seven, and eight), and were employed to determine the impact of group and time status on the alterations in descriptive outcomes. Other parameters were analyzed using one‐way ANOVA. Post hoc testing was completed with Tukey's test, and significance of differences was taken at the level of *p* < .05. Calculations were performed using SPSS software version 20 and the significance level was considered at *p* < .05.

## 3 RESULTS

### 3.1 Ursolic acid and exercise training change weight and blood glucose

**FIGURE 2:**
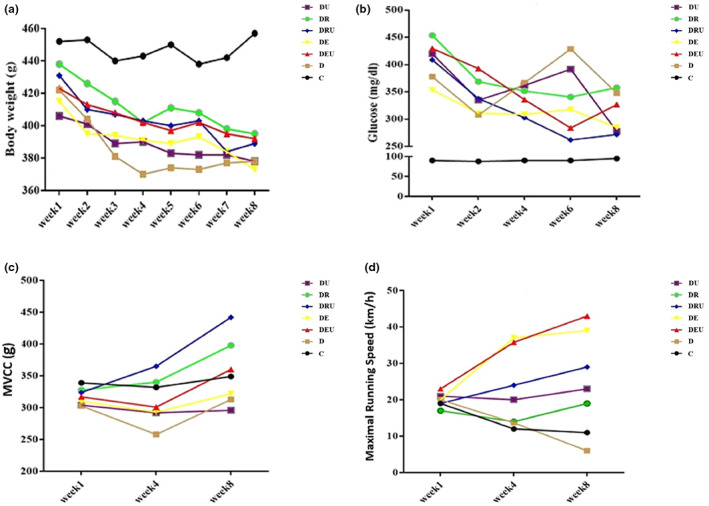
Body weight (a), blood glucose (b), maximal voluntary carrying capacity (MVCC) (c) and maximal running speed (d) changes following 8‐weeks exercise training with or without ursolic acid supplementation. C, Control; D, Diabetic; DE, Diabetic + Endurance Training; DEU, Diabetic + Endurance Training + Ursolic Acid; DR, Diabetic + Resistance Training; DRU, Diabetic + Resistance Training + Ursolic Acid; DU, Diabetic + Ursolic Acid.

The repeated‐measures ANOVA results did not show any significant interaction effect among supplement, time, and training, on the body weight (*p* = .994, *ηp* ^2^ = .024) and the glucose (*p* = .0859, *ηp* ^2^ = .031) following the 8 weeks of interventions. But the glucose showed a significant interaction effect between time and training (*p* < .001, *ηp* ^2^ = .297) (Figure 2).

### 3.2 Ursolic acid and exercise training improve MVCC and maximal running speed

A significant interaction effect between training and time (*p* = .0001, *ηp* ^2^ = .434) was observed for the MVCC.

Considering the significant level of interactive effect among supplement, time, and training (*p* = .024, *ηp* ^2^ = .212), a significant difference in VO_2max_ was observed between the research groups in different weeks. As well, the maximal running speed showed a significant effect time and training and time and supplement. In the first week, there is no significant difference between the groups in terms of VO_2max_. But in the fourth and eighth weeks, Diabetic + Endurance and Diabetic + Endurance + Ursolic acid groups had a significant increase compared to the control and diabetic groups. Also, the interactive effects of supplement and time (*p* = .002) and exercise and time (*p* = .001) were significant (Figure 2).

### 3.3 Ursolic acid and exercise training improve serum lipid profile

**FIGURE 3:**
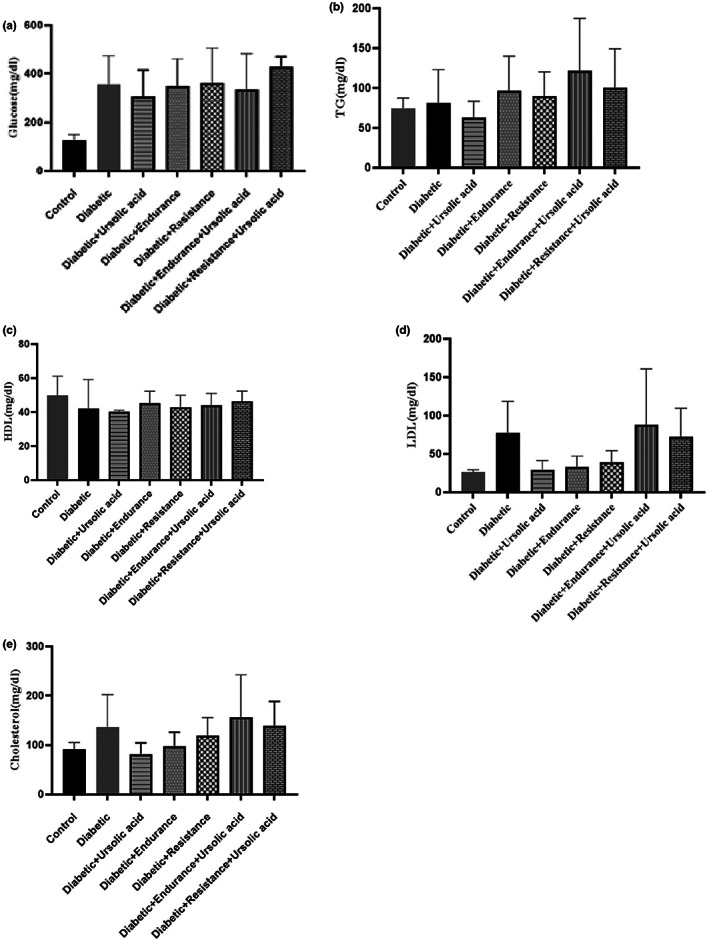
Serum lipid profiles and insulin changes following 8‐weeks resistance and endurance training with or without ursolic acid supplementation.

In this part, despite the decrease in TG, LDL, and cholesterol in some groups compared to the diabetic group, in none of the cases were the changes significant. Also, the highest decrease in glucose compared to the diabetic group was in the groups of Diabetic + Ursolic acid and Diabetic + Endurance + Ursolic acid, however, no significant change was observed. Similarly, HDL in the groups of Diabetic + Endurance, Diabetic + Resistance and Diabetic + Endurance + Ursolic acid, and Diabetic + Resistance + Ursolic acid, despite its increase compared to the diabetic group, was not significant (Figure 3).

### 3.4 Ursolic acid increases SIRT1 levels

#### 3.4.1 Protein levels of SIRT1–eNOS axis changes in study groups

**FIGURE 4:**
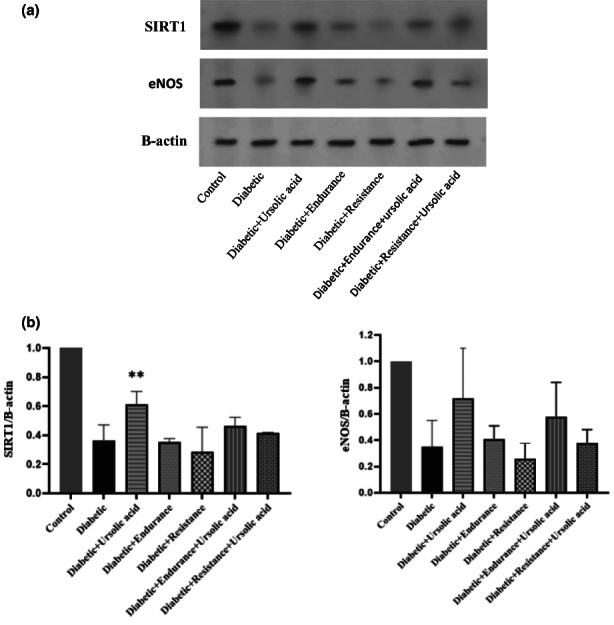
Expression changes of eNOS (endothelial nitric oxide synthase) and SIRT1 (Sirtuin 1) after treatment with exercise training with or without Ursolic acid: (a) western blot bands of SIRT1 and eNOS. B‐actin was used as the control for both eNOS and SIRT1. (b) Column chart of western blot band intensities. All data repeated in triplicate and quantitation for western blot bands was performance by Image J. *p < .05, **p < .01 (in comparison with Diabetic).

According to the results of one‐way ANOVA, the increase in SIRT1 in the Diabetic + Ursolic acid group compared to the diabetic group is significant (*p* = .003), as well as in the groups Diabetic + Endurance + Ursolic acid and Diabetic + Resistance + Ursolic acid compared to the diabetic group, although it was not significant (Figure 4).

The results of correlation test show that there is a significant linear relationship between SIRT1 and eNOS so that with increasing or decreasing SIRT1, eNOS also increases or decreases, but for eNOS, no significant change was observed in any of the groups (*r* = .670, *p* < .001).

### 3.5 Ursolic acid and exercise training reduce vascular oxidative stress

Malondialdehyde (MDA) was decreased in Diabetic + Ursolic acid and Diabetic + Endurance + Ursolic acid groups compared to the diabetic group, but the decrease was not significant. An increase in the amount of SOD antioxidant enzyme was also observed in all groups except the Diabetic + Resistance group compared to the diabetic group, although this increase was not significant. For TAC, the two groups Diabetic + Ursolic acid and Diabetic + Endurance + Ursolic acid were slightly increased compared to the group.

**FIGURE 5:**
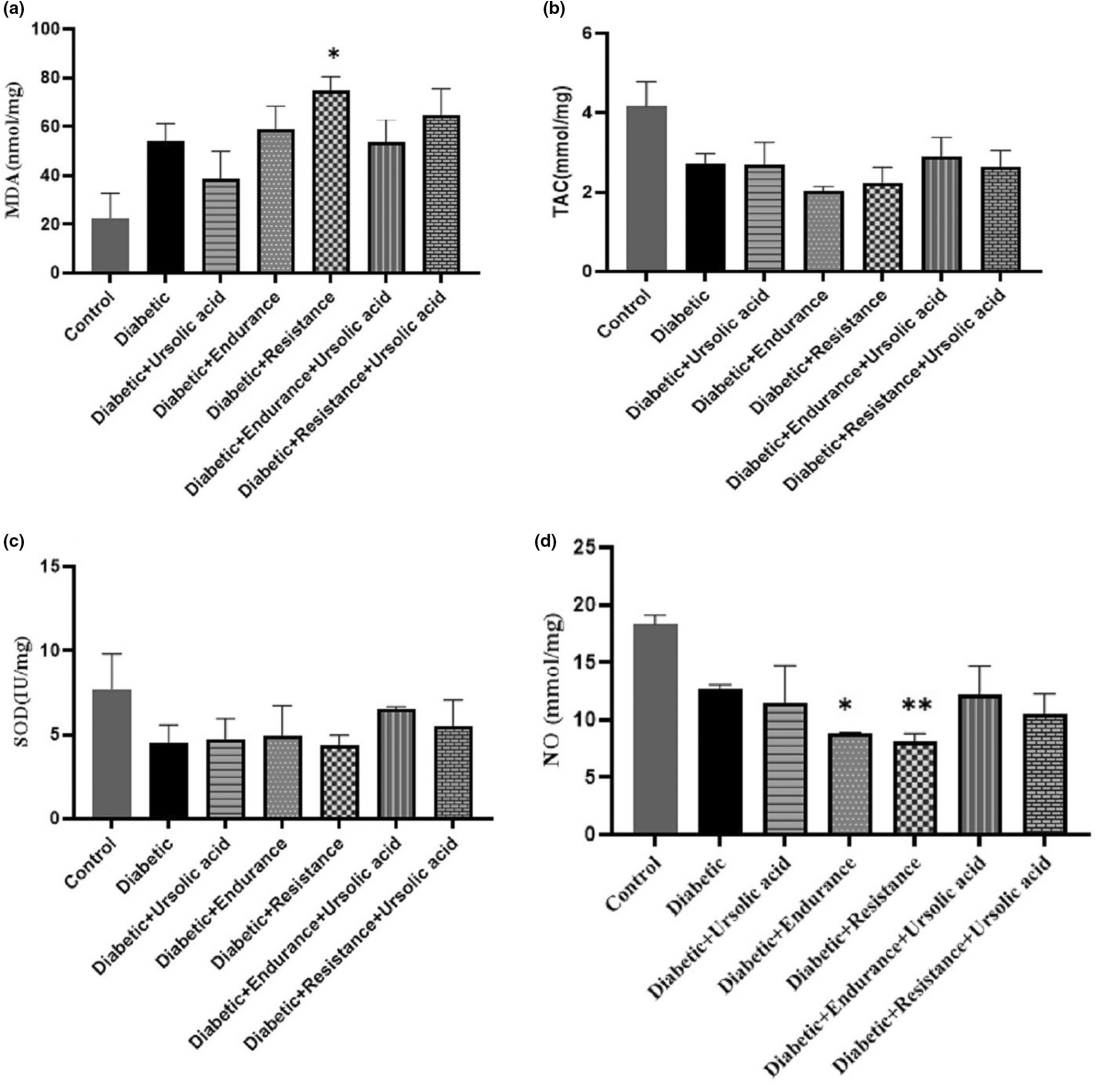
Antioxidant indices following 8‐weeks exercise training with or without ursolic acid. Malondialdehyde (MDA) (a), Total antioxidant capacity (TAC) (b), Superoxide dismutase (SOD) (c) and Nitric oxide (NO) (d) changes following 8‐weeks exercise training and ursolic acid. *: difference with diabetic group *p* < .05, and **: difference with diabetic group *p* < .01.

Finally, it was observed that the amount of NO in the groups of Diabetic + Endurance (*p* = .031) and Diabetic + Resistance (*p* = .007) was significant compared to the Diabetic group. (Figure 5).

## 4 DISCUSSION

Understanding how endothelial–vascular cells sense and respond to distinct training modalities plus UA supplementation has been thus far highly researched in aged diabetic animals. In the present study, the effects of chronic oral UA supplementation, alone and in combination with endurance/resistance training, on vascular aging signaling proteins such as the SIRT1–eNOS axis and the OS indices were aimed in old male Wistar rats with HFD/STZ‐induced T2D. All study data showed that resistance/endurance training plus the UA consumption partially reversed the levels of MDA, NO, as well as TAC in old male Wistar rats with HFD/STZ‐induced T2D. The findings in the present study also revealed that a combination of UA and training did not improve serum glucose, HDL‐C, and TG concentration as vascular aging biomarkers (Kiliç et al., 2021; Walter, 2009; Yu et al., 2019) following 8 weeks of intervention in old male Wistar rats with HFD/STZ‐induced T2D.

Overall, there are some trials evaluating the impact of different training modalities and/or antiaging/diabetes supplements such as UA on major hallmarks of EC senescence (Asmis et al., 2019; Castro et al., 2015; Deckx et al., 2016; Ross et al., 2016; Tanaka, 2019; Ullevig et al., 2011; Zeller et al., 2012).

Therefore, HFD/STZ‐induced T2D aged animals are susceptible to atherosclerosis. In this study, UA supplementation alone was not a potent inhibitor of vascular aging in the HFD/STZ‐induced T2D old animals. One possible reason was that UA could reduce in the flux of the reactive oxygen species (ROS) in monocytes that would be essential for oxidation, mitogen‐activated protein kinase phosphatase 1 (MKP‐1) inactivation, as well as S‐glutathionylation of protetin, but, UA chemical structure illustrated that it would not be readily oxidized and hence there would be a poorer scavenger of ROS (Asmis et al., 2019). Hence, in this study, lack of protection of vascular against OS induced dysfunctions involved in ineffective antivascular aging properties of UA. Another possibility was that the given dose of UA did not modulate the expression of cytokines related to T‐helper 1–2 cells that had possibly influenced the pro‐atherogenic T‐cell responses (Ahmad et al., 2006). However, UA could not reduce food intake and weight gain. It seems that UA may not enhance the metabolic rate of this HFD/STZ‐induced T2D model. In the present model, UA did not affect the lipid profile or the blood glucose levels. Nonetheless, physiologically relevant doses of UA were injected.

It was established in this study that oral 0.5% UA supplementation plus exercise training can prevent vascular aging biomarkers in a HFD/STZ‐induced T2D model.

Ursolic acid (UA) supplementation could enhance the SIRT1 expression in the vein ECs. Jiang et al. (2016) had also shown that 10 weeks of UA supplementation following high‐fat diet had improved serum lipid profile, serum antioxidant status, and morphology of the aorta in the human umbilical vein ECs (HUVECs) (Jiang et al., 2016). Chan et al. (2018) had shown that a long‐term 14‐week aerobic training (60 min per day) for 5 days/per week had improved atherosclerosis status by activating the SIRT1 and inhibiting the OS in the C57BL mice (Chan et al., 2018). Recently, Donniacuo et al. (2019) had established that long‐term moderate training had diminished the apoptosis of cardiac myocytes and had consequently promoted SIRT1 nuclear localization (Donniacuo et al., 2019).

One possibility was that there was a strong correlation between obesity, body mass index (BMI), and SIRT1 gene polymorphisms (Clark et al., 2012; Zillikens et al., 2009). As well, SIRT1 expression was nutrient‐sensitive (Martins et al., 2012). Therefore, the HFD/STZ‐induced T2D rat models in the present study had no significant responses to combined UA supplementation and training considering the SIRT1–eNOS axis. Contrary to these results, Lin et al. had illustrated that resveratrol treatment could increase the beneficial impact of training in aged rat heart models.

It has been shown that training combined with the use of natural antioxidants improves antioxidant system (Mason et al., 2020). UA can further increase the expression of endothelial NOS, reduce nicotinamide adenine dinucleotide phosphate (NADPH) oxidase expression, increase the potency of reversing eNOS uncoupling, and prevent damage to ECs (Förstermann & Li, 2011; Woźniak et al., 2015). It seems that UA plus different types of training modalities possess strong antioxidant properties and this bioactive free radical scavenger compound has potentials to remove free radicals, which is helpful in maintaining the ROS levels (Do Nascimento et al., 2014; Saad et al., 2015). SOD is also considered as an enzyme that regulates the production of oxygen (O_2_) and prevents oxidative damage in the cells.

The findings in the present study revealed that a combination of UA and training did not improve vascular aging biomarkers such as serum glucose, HDL‐Cs, and TG concentration, content following 8 weeks of intervention, which were not consistent with the results reported by Jang et al. (2010) wherein the UA consumption had improved lipid profiles, glucose utilization, and glycogen storage in the liver in STZ‐induced diabetic mice (Jang et al., 2010). In another study, Jayaprakasam et al. (2006) had found that 8 weeks of UA plus anthocyanin supplementation (500 mg/kg of the high‐fat diet) had ameliorated glucose tolerance in the C57BL/6 mice, receiving high‐fat diets.

The discrepancy between the results of the present study and the ones reported by Jayaprakasam et al. (2006) may be attributable to the difference in the compounds other than UA. The study by Jayaprakasam et al. (2006) had accordingly shown that improving glucose status might be associated with UA's ability to enhance insulin levels, which was not established in the present study with a combination of UA plus training, although it did not change the body weight compared with the D group. It seems that old animals having HFD/STZ‐induced T2D might have become insulin‐resistant, and UA plus training intervention has failed to decrease the blood glucose levels.

The lipid profile in the UA plus training‐treated HFD/STZ‐induced‐T2D rats was similar to that of Diabetic. It had been established that UA could moderately decline in the liver lipid deposition (Xia et al., 2005).

Another possibility discussing that the consumption of UA plus training did not improve vascular aging was that the side effects of UA of a supraphysiological dose were capable of making platelets more susceptible to aggregation (Kim et al., 2014), which was not noticed for antivascular aging in ECs. Indeed, antiplatelet aggregation of UA supplementation was not enough to exert a significant effect. In addition, it had been shown that the UA solubility was too low to be applied to platelets. Instead, it has been supposed that UA supplementation might make platelets sensitive to stimuli (Kim et al., 2014). Furthermore, circulating UA concentrations were not measured in the present study, but an oral dose of 250 mg UA per kg of body weight rat/day was used, which could be lower than the concentration enough for physiological effects in vascular aging biomarkers.

Considering MDA as an endogenous product of both enzymatic and oxygen radical‐induced lipid peroxidation (Niedernhofer et al., 2003). Somova et al. (2003) had found that UA had increased glutathione peroxidase and SOD levels in insulin‐resistant rats (Somova et al., 2003). In another study, it had been established that UA had boosted SOD activity and had diminished MDA formation in PC12 cell line (Tsai & Yin, 2008), so that decreased levels of MDA indicated that the UA could inhibit lipid peroxidation. Overall, this study showed that the use of UA in combination with exercise could increase vascular antioxidant capacity in HFD/STZ‐induced T2D‐induced elderly Wistar rats in some cases with the interventions studied, although this change was not significant. In addition to comparing the combined effects of UA and different exercise protocols, the results showed that no significant differences were seen in the variables‐related vascular aging following resistance and endurance training alone. Precise comparisons between endurance and a resistance exercise protocol on vascular aging biomarkers are scarce. This can strengthen the argument that resistance and endurance training does not affect the vascular function, and moderate‐intensity endurance/resistance training causes no significant change in arterial stiffness of old male Wistar rats with HFD/STZ‐induced T2D. This indicates that the impact of endurance/resistance training on vascular function of old male Wistar rats with HFD/STZ‐induced T2D is not as great, or the resistance training performed was not effective at changing vascular function biomarkers, which could be the same with many of the studies showing no change in vascular function (Tabaie et al., 2021). Different studies showed no significant changes in vascular function following resistance training (Augustine et al., 2014; Croymans et al., 2014; Kujawski et al., 2018). Furthermore, it is possible that there are beneficial effects of endurance exercise training on central arterial compliance following shorter duration of exercise training than that of the present study (Shibata & Levine, 2012). The discrepancy could be explained by the fact that indexes for vascular aging used in these previous studies involved systemic arterial compliance, which are affected by both structural and functional components of arterial compliance (Shibata & Levine, 2012). Indeed, endurance exercise training would not be most effective at improving the vascular aging biomarkers and could not possibly affect structural components when insulin concentration is not improved (Lee et al., 2018).

There are, however, some limitations that should be noted. The methodology utilized in this study allowed for the analysis of only a few vascular aging boimarkers. Furthermore, only aged rats were studied for different types of exercise training protocols. Future work should thus compare the effects of these protocols on vascular aging biomarker levels, as well as their correlations between aged and young animals. Finally, we did not evaluate vascular aging directly through aortic stiffness measured by the pulse wave velocity method.

In conclusion, this study analyzed the effects of concurrent treatment using UA and resistance/endurance training to improve vascular aging biomarkers using an aged HFD/STZ‐induced T2D animal model. The effect of UA alone on vascular aging biomarkers could not be confirmed; however, positive effects could be confirmed for UA supplementation plus resistance/endurance training. Further studies will be needed to verify the differences in the concentration of UA and the need to additionally analyze the relevant signaling pathway to confirm a mechanism for use of UA as a supplement of exercise training. This study is meaningful in that UA was identified to have a possible use as a supplement of exercise training for reduction of vascular aging. The practical significance of this study was providing insight into a new way to combat vascular aging in elderly patients with type 2 diabetes. It is quite conceivable that supplementation of UA (is usually found in the fruit peels and stem bark as secondary metabolites) plus exercise training may be a viable therapeutic strategy for treatment of diseases including vascular aging in elderly type 2 diabetes patients.

## CONFLICT OF INTEREST

There was no competing interest.

## ETHICS STATEMENT

The local Ethics Committee for Laboratory Animals of Shahrekord University (IR.SKU.REC.1399.001, ethics.research.ac.ir) approved the study. All methods were performed in accordance with the relevant guidelines and regulations. The experiment was conducted in accordance with the ARRIVE guidelines (Animal Research) for the care and use of research animals.

## Data Availability

The data that support the findings of this study are available from the corresponding author upon reasonable request.
